# Bimodal Activation of Different Neuron Classes with the Spectrally Red-Shifted Channelrhodopsin Chimera C1V1 in *Caenorhabditis elegans*

**DOI:** 10.1371/journal.pone.0046827

**Published:** 2012-10-03

**Authors:** Karen Erbguth, Matthias Prigge, Franziska Schneider, Peter Hegemann, Alexander Gottschalk

**Affiliations:** 1 Buchmann Institute for Molecular Life Sciences, Goethe-University, Frankfurt, Germany; 2 Institute of Biochemistry, Goethe-University, Frankfurt, Germany; 3 Institute of Biology (Experimental Biophysics), Humboldt-University, Berlin, Germany; Yale School of Medicine, United States of America

## Abstract

The *C. elegans* nervous system is particularly well suited for optogenetic analyses of circuit function: Essentially all connections have been mapped, and light can be directed at the neuron of interest in the freely moving, transparent animals, while behavior is observed. Thus, different nodes of a neuronal network can be probed for their role in controlling a particular behavior, using different optogenetic tools for photo-activation or –inhibition, which respond to different colors of light. As neurons may act in concert or in opposing ways to affect a behavior, one would further like to excite these neurons concomitantly, yet independent of each other. In addition to the blue-light activated Channelrhodopsin-2 (ChR2), spectrally red-shifted ChR variants have been explored recently. Here, we establish the green-light activated ChR chimera C1V1 (from *Chlamydomonas* and *Volvox* ChR1′s) for use in *C. elegans*. We surveyed a number of red-shifted ChRs, and found that C1V1-ET/ET (E122T; E162T) works most reliable in *C. elegans*, with 540–580 nm excitation, which leaves ChR2 silent. However, as C1V1-ET/ET is very light sensitive, it still becomes activated when ChR2 is stimulated, even at 400 nm. Thus, we generated a highly efficient blue ChR2, the H134R; T159C double mutant (ChR2-HR/TC). Both proteins can be used in the same animal, in different neurons, to independently control each cell type with light, enabling a further level of complexity in circuit analyses.

## Introduction

Optogenetic tools of microbial origin have been effectively adopted to control activity of neurons and other cells by light in a number of model systems, and are widely used, also in *Caenorhabditis elegans* [1]–[4]. First of all, the blue light-driven cation channel Channelrhodopsin-2 (ChR2), derived from an eye-spot photoreceptor of *Chlamydomonas reinhardtii* [5], can be used to photodepolarize neurons or other excitable cells like muscles [6]–[9]. Other optogenetic tools include the yellow-light driven chloride importer Halorhodopsin from *Natronomonas pharaonis* (NpHR), which is used for photohyperpolarization [10]. NpHR can be co-expressed with ChR2. The two proteins can be controlled independently, due to the distinct action spectra. Further inhibitory optogenetic tools are rhodopsins that act as proton exporters, like Mac or Arch, which are driven by blue-green and green-yellow light, respectively, and that can also be used as hyperpolarizers, either concomitantly with, or independently of, ChR2 [11]–[13], In addition, light-driven enzymes such as photoactivated adenylyl cyclase (PAC) were introduced for optical manipulation of the cAMP level [14]–[17].

The possibility to co-express optogenetic tools and to independently activate them using different colors of light enables multimodal investigation of neural networks in *C. elegans* [10], [13], [18]. For photo-depolarization, however, there were until recently only several variants of the blue-light driven ChR2 available [19]–[21]. Thus, spectrally red-shifted photodepolarizers would be desirable, as they would allow to independently, yet concomitantly, activate two different sets of neurons. To achieve excitatory optogenetic manipulation in the green to red range of the optical spectrum, different approaches were taken by several groups. They either made use of red-shifted absorption properties of ChRs exhibited by other microbial sources *per se,* like *Volvox carteri* (VChR1) [22], [23] or *Mesostigma viride* (mChR1) [24]. Alternatively, the action spectrum of the well expressing ChR2 could be color shifted by substitution of the chromophore all-*trans*-retinal (ATR) by spectrally distinct retinal analogues with variable backbone substitutions or altered π-electron system [25]. This can be achieved in *C. elegans*, where retinal is added exogenously, along with the diet [7], but hardly in vertebrates that contain high levels of intrinsic ATR. Moreover, ChR properties can be altered by site-directed mutagenesis [19], [20] which might also affect absorption properties [26].

Here, we surveyed a number of channelrhodopsin variants for their potential use as efficient green-light activated depolarizers in *C. elegans*. Ultimately, we were successful when using a previously reported chimeric ChR fusion protein from *Chlamydomonas* ChR1 and *Volvox* VChR1 including certain point mutations (E122T and E162T; yielding C1V1-ET/ET) [23]. This protein enabled dual color excitation together with ChR2s in the *C. elegans* neuromuscular and nervous systems. Even though C1V1-ET/ET showed a significantly red-shifted action spectrum, it was still moderately efficient in the blue range of the spectrum, and could further photostimulate cells at low light intensities. Thus, to achieve truly independent bimodal two color activation, we had to combine C1V1-ET/ET with a “high-efficiency” blue-light activated ChR2. To this end, we combined the two “gain of function” variants T159C and H134R (yielding ChR2-HR/TC), to achieve blue-light activation with low light intensities that would not co-activate C1V1-ET/ET. We demonstrate the utility of C1V1-ET/ET and ChR2-HR/TC in body wall muscles, and in combination in different neuron types, allowing independent and concomitant activation with blue and green light, respectively, in live *C. elegans*.

## Methods

### *C. elegans* Culture and Transgenic Strains

*C. elegans* strains were grown at 20°C in the dark on standard nematode growth medium (NGM) plates in the presence of OP50-1 bacteria and all-*trans* retinal (ATR), as previously described [27]. Transgenic strains were obtained by standard procedures, i.e. microinjection of DNA into the gonads of either *lite-1(ce314); lin-15(n765ts)* nematodes, together with a *lin-15* containing rescue plasmid (80 ng/µl), or into strain ZX388 (see below) [27], or into wild type (N2) animals together with pCFJ90, a *pmyo-2::mCherry* marker plasmid [28], at 2 or 2.5 ng/µl. For ZX1163 and ZX1165, 1kb+ DNA ladder (Fermentas) was co-injected at 300 and 80 ng/µl, to generate appropriate controls for the following transgenic strains: In ZX1283, ZX1164 and ZX1165, *punc-47::ChR2(H134R; T159C)::YFP* was injected at 80 ng/µl, in ZX1283, ZX1162 and ZX1163, *punc-17::C1V1(E122T; E162T)::YFP* was injected at 300 ng/µl. In some strains, *rol-6d* was the co-injection marker, used at 80 ng/µl. The following transgenic strains were generated and/or used:

**ZX299:** lin-15(n765ts^−^); zxEx22[pmyo-3::ChR2(H134R)::YFP; lin-15^+^] [7], **ZX388:** lin-15(n765ts^−^); zxIs3[punc-47::ChR2(H134R)::YFP; lin-15^+^] [27], **ZX976**: N2; zxEx512[pmyo-3::C1V1::YFP; pmyo-2::mCherry], **ZX978**: lite-1(ce314); lin-15(n765ts); zxEx514 [pmec-4::C1V1::mCherry; pglr-1::flox::CpV-D3; pgpa-14::nCre; lin-15^+^], **ZX980:** N2; zxEx516[pmyo-3::C1V1(E122T; E162T)::YFP; pmyo-2::mCherry], **ZX981:**lin-15(n765ts^−^); zxIs3[punc-47::ChR2(H134R)::YFP; lin-15^+^]; zxEx517[pmyo-3::C1V1(E122T; E162T)::YFP; pmyo-2::mCherry], **ZX982:** lin-15(n765ts^−^); lite-1(ce314); zxEx518[pmec-4::C1V1(E122T; E162T)::mCherry; pglr-1::loxp-STOP::CpV-D3; pgpa-14::nCre; lin-15^+^], **ZX1405:** N2; zxEx[pmyo-3::ChEF(C1C2 5-2)::YFP; rol-6d], **ZX1406:** N2; zxEx[pmyo-3::VChR1hum::YFP; rol-6d], **ZX1407:** N2; zxEx[pmyo-3::VChR1(trunc)::YFP; rol-6d], **ZX1408:** N2; zxEx[pmyo-3::VChR1short::YFP; rol-6d], **ZX1409:** N2; zxEx[pmyo-3::ChR2_signal-peptide::VChR1short::YFP; rol-6d], **ZX1410:** N2; zxEx[pmyo-3::C1V1 (E122T/V196I/G197A)::YFP; pmyo-2::mCherry], **ZX1411:** N2; zxEx[pmyo-3::V1C2 2-2-3::YFP; rol-6d], **ZX1162:** N2; zxEx532[punc-17::C1V1(E122T; E162T)::YFP; pmyo-2::mCherry], **ZX1163:** N2; zxEx533[punc-17::C1V1(E122T; E162T)::YFP; 1 kb+ DNA ladder, pmyo-2::mCherry], **ZX1164:** N2; zxEx534[punc-47::ChR2(H134R; T159C)::YFP; pmyo-2::mCherry], **ZX1165:** N2; zxEx535[punc-47::ChR2(H134R; T159C)::YFP; 1 kb+ DNA ladder, pmyo-2::mCherry], **ZX1166:** N2; zxEx536[pmyo-3:: ChR2(T159C)::YFP; pmyo-2::mCherry], **ZX1167:** N2; zxEx537[pmyo-3:: ChR2(H134R; T159C)::YFP; pmyo-2::mCherry], **ZX1283:** N2; zxEx531[punc-17::C1V1(E122T; E162T)::YFP; punc-47::ChR2(H134R; T159C)::YFP; pmyo-2::mCherry], **AQ2334:** lite-1(ce314); ljIs123[pmec-4::ChR2; punc-122::RFP].

### Molecular Biology

Chimeric ChR variants were generated from synthetic human codon-adapted COP3, COP4 (GenBank EU714030.1), or VOP3 by overlap extension PCR as described elsewhere [29]. The resulting PCR fragments were cloned in-frame into pECFP-N1 using XbaI/BamHI. Point mutations were generated with Quikchange (Agilent Technologies, Palo Alto, CA).

*pmyo-3::ChR2(H134R)::YFP* [7] was used as a template to sub-clone VChR1hum, VChR1 (trunc), VChR1 short, VChR1short SP, ChEF (C1C2 5-2), C1V1 and C1V1 ET/VI/GA preceding YFP, by using BamHI and SfoI/NotI+Klenow fill-in, BamHI and NotI, BamHI and SacII, Bsu36I and SacII, BamHI and DraIII and BamHI and KpnI restriction sites, respectively, to insert the respective DNA behind the p*myo-3* promoter. Coding sequences of VChR1trunc (amino acids 1–283), VChR1 short (amino acids 1–300) and VChR1 short SP were previously amplified by PCR from pGCR-1_S1/3::VChR1 using Primers oKE10+11, oKE10+21 or oKE19+20 and ligated using In-Fusion Cloning Kit (Clontech), resulting in plasmids pKE8 (*pmyo-3::VChR1(trunc)::YFP*), pKE12 (*pmyo-3::VChR1short::YFP; rol-6d*) and pKE11 (*pmyo-3::ChR2SP::VChR1short::YFP*). pKE10 (*pmyo-3::VChR1hum::YFP*) and pKE13 (*pmyo-3::ChEF (C1C2 5-2)::YFP*) were obtained from pGA-VChR1hum and pGEM ChR1-2_5/2_Chlamy. V1C2 2-2-3 was subcloned from plasmid pv2-c2-v3 into *StuI* and *SacII* restriction sites of pKE10 yielding pKE14 (*pmyo-3::V1-C2 2-2-3::YFP*), by In-Fusion cloning. C1V1 and C1V1 ET/VI/GA cDNA were amplified via PCR from plasmids N1.hC1/hV1 and N1.hC1/hV1(E122T/V196I/G197A) using Primers C1V1_up_BamHI and C1V1_low_KpnI and introduced into BamHI and KpnI restriction sites of plasmids *pmyo-3::ChR2(H134R)::YFP* [7] and *pmec-4::ChR2(H134R)::mCherry*. The N1.hC1/hV1(E122T; E162T) plasmid, containing the C1V1(E122T; E162T) cDNA, was used to obtain *pmyo-3::C1V1(E122T; E162T)::YFP* and *pmec-4::C1V1(E122T; E162T)::mCherry* constructs, by subcloning a 231bp piece, containing the mutated sites, into *pmyo-3::C1V1::YFP* and *pmec-4::C1V1::mCherry* vectors, using StuI and AarI restriction sites. *pmec-4::C1V1(E122T; E162T)::mCherry* was then used as a template to insert the E122T; E162T double mutation in the NaeI and BsiWI restriction sites of plasmid *punc-17::C1V1::mCherry*, leading to *punc-17::C1V1(E122T; E162T)::mCherry. Pglr-1::loxp-STOP::CpV-D3* was obtained by first opening *pglr-1::CpV-D3* via XbaI restriction and inserting loxp-*STOP*, removed from plasmid pNP165 (a gift from Navin Pokala) with NheI, into the vector, preceding CpV-D3. To construct *pmyo-3::ChR2(T159C)::YFP* and *punc-47::ChR2(T159C)::YFP*, a PCR product was amplified from *pChR2(T159C)* (a gift from G. Nagel), using primers oKE79 and oKE80 and inserted into *pmyo-3::ChR2::YFP* and *punc-47::ChR2(H134R)::YFP* with Bsu36I and DraIII restriction sites. To generate *pmyo-3::ChR2(H134R; T159C)::YFP*, two PCRs were performed with oKE79+81 and oKE80+82 from *pmyo-3::ChR2(H134R)::YFP* and *pChR2(T159C)*, respectively, leading to fragments carrying H134R and T159C separately, that were then fused and inserted in the *pmyo-3::ChR2(H134R)::YFP vector*. *pChR2(H134R; T159C)* from *pmyo-3::ChR2(H134R; T159C)::YFP* was then inserted into *punc-47::ChR2(T159C)::YFP* via Bsu36I and BsiWI restriction sites, resulting in *punc-47::ChR2(H134R; T159C)::YFP.*

### Primers Used

oKE10: TCCATCTAGAGGATCCATGGATTATCCCGTTGCTCGGTCT

oKE11: ACCATGGTGGCGGCCGCGGGTACACGCAGGTAGTTGCCGAGAA

oKE19: CCTGAGGCCTGCATGCCGCGCGGCCAGTGC

oKE20: CCGCGGCGTGATTAGTCACGAATGCATACTTGGC

oKE21: CGCCGCCGCGGCGTCCTCCTCTTCGGCCACAAGAGTC

oKE79: GTCGTCAATGGCTCTGTACTTGTG

oKE80: AGAGCCAAGCCATGCCAGTC

oKE81: GCCAATATCAGACACAAGCAGACC

oKE82: GGGTCTGCTTGTGTCTGATATTGG

C1V1_up_BamHI: GGCCGGGATCCATGAGCAGACGGCCCTGGCTGC

C1V1_low_KpnI: GCCGGGGTACCGGTCTGCTGGCGTACTTGGCGGTGC

### HEK Cell Recording and Data Analysis

HEK293 cells were cultured as described [30] and seeded onto cover slips at a concentration of 0.175×10^6^ cells/ml and supplemented with 1 µM *all trans*-retinal. Transient transfection was performed using Fugene 6 (Roche, Mannheim, Germany) 20–28 h before measurement. Signals were amplified using a Heka EPC7 and DigiData1400. For recording wavelength dependency a light guide from a Polychrome V unit (TILL Photonics, Planegg, Germany) was mounted on the epi-illumination port of an Olympus IX70 microscope resulting in a final light intensity between 0.05–0.23 mW/mm^2^ for 380–650 nm on the coverslip. Action spectra as shown in Fig. S4 were recorded at 50% of the maximum light intensity with a bandwidth of 7 nm. The Polychrome V Unit was controlled via the Tillvision Software (TILL Photonics, Planegg, Germany) synchronized with the pClamp Software. The standard external solution contained [mM]: 140 NaCl, 2 CaCl_2_, 2 MgCl_2_, 2 KCl with 10 HEPES (pH 7.2). The standard internal solution contained [mM]: 110 NaCl, 10 EGTA, 2 MgCl_2_, 1 CaCl_2_, 5 KCl and 10 HEPES. Data was analyzed using TillVision Software or pClamp10.1 and further processed by SigmaPlot and Adobe Illustrator. Action spectra were linearly normalized on the light intensity at each wavelength.

### Fluorescence Microscopy

Expression of YFP fusion proteins in *C. elegans* body wall muscle cells or ChR2-HR::YFP in GABAergic motor neurons was analyzed on a Zeiss Axiovert 200 or a Zeiss Axio Observer microscope, equipped with a 100 W HBO mercury lamp, 40× or 100× objective, and a YFP filterset (F41-028, AHF Analysentechnik). Images were obtained with a Zeiss Axiocam MRm and Axiovision software or CoolSNAP HQ2 camera (Roper Scientific) and MetaVue software. In ZX1283 animals, expression of ChR2(H134R/T159C)::YFP and C1V1(E122T; E162T)::mCherry were analyzed with a 60× objective under an Eclipse TE2000E/C1 Plus confocal laser scanning microscope system (Nikon). A 488 nm single line 10 mW Argon laser and a 543 nm 1.5 mW HeNe laser were used to excite YFP and mCherry, respectively. Saturation, dark and gamma levels of fluorescence images were adapted in relation to the laser power in order to reduce photobleaching of the fluorophores. Image post-processing, if at all, was restricted to cropping, rotation, brightness and contrast by ImageJ (NIH) as well as saturation, dark and gamma levels by EZC1 (Nikon).

### *C. elegans* Behavioral Experiments

Transgenic L4 larvae were placed on fresh ATR plates one day prior to the assay. For sets of experiments including C1V1-ET/ET, young adult animals were observed for reactivity to green or blue light under an MZ16F dissection scope (Leica) at least two hours before the assay and well responding animals were transferred to freshly seeded ATR plates. For assays, animals were individually picked on NGM plates without bacterial food. Assays were performed under a 10× objective on a Zeiss Axiovert 200 or a Zeiss Axio Observer microscope, with a 100 W HBO mercury lamp, F38–651 beamsplitter (AHF Analysentechnik) combined with narrow BP excitation filters 400±10 nm, 420±10 nm, 436±10 nm, 458±10 nm, 480±10 nm, 500±10 nm, 520±10 nm, 540±10 nm, 568±10 nm, 580±10 nm and 600±10 nm (Edmund Optics). Light intensity was adjusted before each assay with the lamp voltage control and neutral density filters (Zeiss), using a PM 100 power meter with S120UV Sensor (Thorlabs).

**Figure 1:**
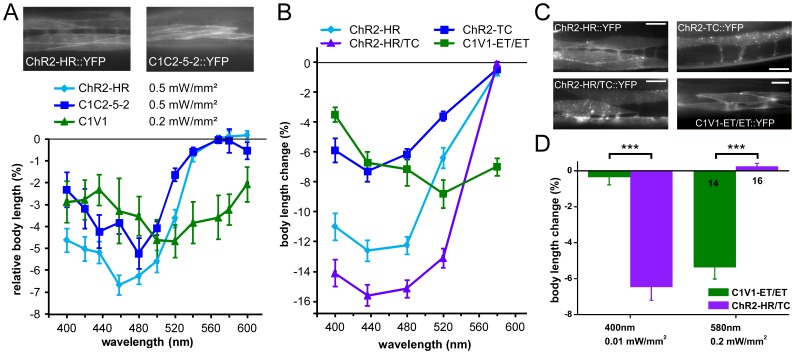
Survey of various ChR variants for red-shifted excitation in *C. elegans* muscle. **A)** Upper panel, fluorescence micrographs. ChR2(H134R)::YFP (ChR2-HR), C1C2 5-2::YFP and C1V1::YFP (for expression pattern, see Fig. S5A), were expressed in body wall muscle cells and photostimulated (lower panel; light intensities and wavelength of band pass filters used are indicated). The resulting body length changes (contractions) were measured relative to the initial length of unstimulated animals. N = 7–24 animals were used for each experiment. **B)** ChR2-HR, ChR2-TC, ChR2-HR/TC and C1V1-ET/ET were expressed in muscles cells and the resulting body length changes in response to 0.2 mW/mm^2^ light at 5 different wavelengths were measured. N = 9–10 animals each. In A and B, absolute contractions (during light) were significantly different from the length before photostimulation for all data points, except for ChR2 and C1C2 at 560 and 568 nm. For a comparison of normalized data of the experiments in A and B, and analysis of statistically significant differences in contractions relative to those evoked by ChR2-HR, see Fig. S3. **C)** Expression patterns of the 4 proteins described in B, each fused to YFP, in body wall muscle cells. Scale bars are 20 µm. **D)** Light-evoked contractions of animals expressing C1V1-ET/ET or ChR2-HR/TC (individually) in body wall muscles were compared at 400 nm (0.01 mW/mm^2^) and 580 nm (0.2 mW/mm^2^). Each protein evokes strong contractions at its near-peak wavelength, but is not co-activated by the respective other wavelength. Number of animals is indicated. In A, B, D, mean body length changes and s.e.m. are displayed. In D, statistically significant differences after two-tailed Student’s t-test are indicated (***p<0.001).

For length measurements, forward moving animals were successively illuminated with different wavelengths and intensities, using a computer-controlled shutter (Sutter Instruments). For sets of experiments including C1V1-ET/ET, movies were recorded beginning 3 or 5 s before and ending 3 or 5 s after the illumination (10fps), using a CMOS camera (DCC1545M, Thorlabs). Body length in each frame was determined using a custom written LabView program [18]. Body length was normalized to the averaged values measured during 1s before illumination. Normalized body lengths measured at the last second of the 2 s illumination period were thereby calculated to the percent change and were then averaged for each worm strain and illumination setting (Fig. S4B). In other experiments, animals were recorded with a Canon Powershot G5 or G9 digital camera with 15 or 30 fps. For most sets of experiments including C1V1, videos were analyzed with automated length extraction software described before [27] and data was analyzed as described above. For action spectra measurements of stains carrying VChR1 variants, ChR2(H134R), from now on ChR2-HR, and ChEF (C1C2-5-2) (Fig. 1A), length was determined manually by extracting selected pictures before and during illumination and extracting worm lengths in pixels by drawing a median through the body length [7]. Data was analyzed by calculating the percentage of body length change in response to illumination for each worm and then averaged.

For escape responses induced by mechanosensory neuron photostimulation, groups of 5 transgenic animals expressing ChR2-HR, C1V1 or C1V1-ET/ET in touch receptor neurons were successively illuminated. The illumination sequence consisted of 5 flashes of 0.5 s, each followed by a 10 s dark period, with 400 or 438 nm light and 568 nm light. Withdrawal responses, or sometimes an abrupt halt, were counted as 1, and no responses within the illumination period were counted as 0, as well as already backward moving animals. The average value for each animal was taken as backing probability (%), and these values were averaged for each genotype and illumination setting.

## Results

### Survey for Potential Function of Green-light Activated Depolarizing Rhodopsins in *C. elegans*

To seek for possibilities to achieve red-shifted excitation via a ChR variant in *C. elegans*, we tested several ChR proteins, chimeras or mutants (Fig. S1): First we tried the green-light activated *Volvox* VChR1, as this has been described for neuronal applications already in 2008 [22]. We expressed this protein in body wall muscle of *C. elegans*, where photoactivation of *Chlamydomonas* ChR2 (CChR2) causes contraction and thus a shrinking of the whole animal [7]. We tested several versions of the native *Volvox* sequence with different length C-termini (“short”, “truncated”), a version in which we replaced the VChR1 signal peptide with the one of CChR2, and a version with human-adapted codon usage (Fig. S1, S2). However, neither of the four VChR1 variants tested could evoke contractions efficiently when illuminated with 540 nm light (in contrast to ChR2-HR), exposed to 460 nm light; Fig. S2B). The proteins expressed comparably weakly, and expression from non-integrated transgenes was rather prone to mosaicisms (Fig. S2A). Thus, VChR1 proved to be not useful in the *C. elegans* background.

Next, we tested several previously described chimeric ChRs from *Chlamydomonas* and *Volvox*, in the *C. elegans* background. The rationale was to potentially combine the beneficial expression properties of CChR2 with the green-shifted action spectrum of VChR1. We tested a CChR1-CChR2 chimera (C1C2-5-2) containing trans-membrane (TM) helices 1–5 from CChR1 and 6–7 from CChR2 (also termed ChEF) and for which a slight red-shift of the photocurrents was reported previously [29], [31], [32]. C1C2-5-2 expressed well in *C. elegans* muscle and evoked efficient muscle contractions. However, it did not markedly shift the action spectrum far enough from the ChR2-HR spectrum due to contribution of the acidic and alkaline isoforms [22], [32] (Fig. 1A; see Fig. S3 for normalized data and significance; see Fig. S4B and Methods for how action spectra in *C. elegans* were measured). Another chimera tested was the V1C2-2-2-3 protein (Fig S1, S5). This is a VChR1/ChR2 chimera (VChR1 TM1-2; CChR2 TM3-4; VChR1 TM5-7), which we expected to contain all sequences important for the red-shift found in VChR1, but with the better expression properties of ChR2. In HEK 293 cells, this protein expressed well, and exhibited a red-shift of its peak to ∼540 nm (Fig. S4A). However, this protein did not express well in *C. elegans* and evoked only minor contractions in body wall muscle (Fig. S5B). Finally, we turned to the chimera C1V1 5-2 (a CChR1 TM1-5; VChR1 TM6-7 protein, in short C1V1 (Fig. 1A, S1, S3, S5). C1V1 expressed moderately well (Fig. S5A), and could evoke body contractions of ∼4% with 520–540 nm light. This is rather weak, when compared to ChR2-HR, which could evoke ∼7–12% contraction at its spectral peak (Fig. 1A, B). The action spectrum of C1V1 showed a peak activity of ∼520 nm. We further tested the possibility to depolarize and activate neurons using C1V1. In mechanosensory neurons, ChR2 photoactivation causes an escape behavior (mostly backward locomotion), as these animals have the sensation of body touch [7]. C1V1 photoactivation also evoked escape behavior in ∼48% of animals tested, with 568 nm light, and only ∼22% responded at 436 nm light (Fig. S6). However, in similar experiments, ChR2-HR evoked responses in ∼84% of animals at 436 nm, and in ∼14% at 568 nm. Thus, a clear green-shift of the action spectra of C1V1 compared to ChR2-HR is apparent, yet C1V1 activity was not overly high in *C. elegans* cells.

We had previously modified C1V1 further with distinct point mutations. These aimed to achieve better expression, but also to reduce a rather pronounced shoulder in the blue range of the spectrum, resulting from the alkaline isoform, which would cause significant activity of C1V1 with blue light (Fig. S4A). These are the triple mutant E122T; V196I; G197A, and the double mutant E122T; E162T (in short C1V1-ET/VI/GA and C1V1-ET/ET). The triple mutant expressed well in *C. elegans* muscle cells, albeit with high mosaicisms, yet no contractions could be evoked (Fig. S5A, B).

### C1V1-ET/ET Activation Evokes Contraction of Muscle Cells in vivo

In contrast, the C1V1-ET/ET double mutant expressed at high levels in body wall muscle cells (transgene *zxEx516[pmyo-3::C1V1-ET/ET::YFP; pmyo-2::mCherry]*) and localized to membranes (Fig. 1C). Thus, we expected, despite the overlap in spectra at the blue end, that ChR2 and C1V1-ET/ET may be activated separately by distinct colors of light at off-peak wavelengths. To compare the functionality and optical compatibility of C1V1-ET/ET with ChR2, we assessed muscle contractions in response to light of different wavelengths in strains expressing either ChR2-HR, or C1V1-ET/ET (Fig. 1B). When exposed to 0.5 mW/mm^2^ of 400 nm light, animals expressing ChR2-HR in muscles displayed ∼11% reduction in body length, respectively, while 580 nm light evoked no contraction (Fig. 1A, B). In contrast, C1V1-ET/ET evoked ∼7% contraction at 580 nm (Fig. 1B). However, exposure to 400 nm light still activated C1V1-ET/ET (∼1 and ∼3–3.5% at 0.1 and 0.5 mW/mm^2^, respectively; Fig. 1B, 3B), due to the short wavelength ß-band of rhodopsin, but this blue response was much smaller than the one evoked by ChR2-HR at this wavelength. At 0.01 mW/mm^2^ of 400 nm light, C1V1-ET/ET showed no notable responses anymore (**Fig. 1D**).

### Photoactivation of C1V1-ET/ET in Mechanosensory Neurons Evokes Escape Behavior

**Figure 2:**
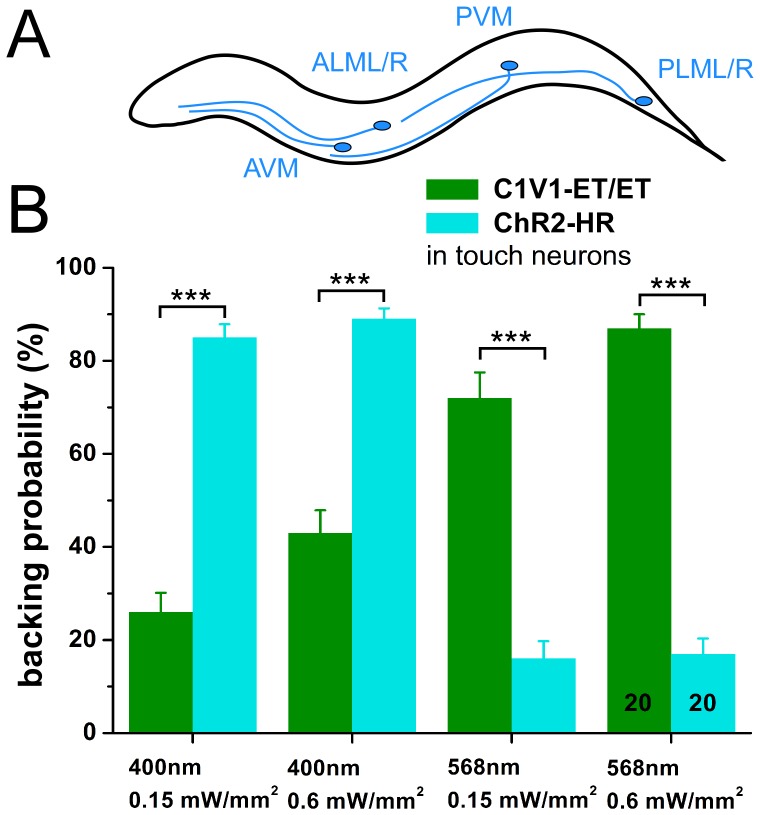
C1V1-ET/ET and ChR2-HR evoke wavelength-dependent activity in mechanosensory neurons. A) The six gentle touch mechanoreceptor neurons, their localization and morphology are shown. **B)** The effectiveness of C1V1-ET/ET and ChR2-HR, expressed in different strains, in evoking behavioral responses in mechanoreceptor neurons (backward escape, reversals) were compared, in response to 400 and 568 nm light of different intensities, as indicated. While C1V1-ET/ET can be specifically activated without co-activation of ChR2-HR at 568 nm, significant co-activation of C1V1-ET/ET at 400 nm is apparent. Displayed is the mean probability of backing in response to blue light stimuli (and s.e.m.), as described in Methods. Number of animals is indicated, and statistically significant differences were determined by two-tailed Student’s t-test, as indicated (***p<0.001).

As described above for C1V1, we tested the ability of C1V1-ET/ET to trigger neuronal function. To this end we expressed C1V1-ET/ET::mCherry in the six gentle touch mechanosensory neurons (Fig. 2A). Animals expressing ChR2-HR in gentle touch neurons were used as a control, and the ability of different light colors to evoke behavior was tested. When illuminated with 568 nm (0.15 and 0.6 mW/mm^2^), C1V1-ET/ET animals showed significantly higher backing probability than did ChR2-HR controls (72 and 87%, compared to 16 and 17%). This proportion was almost inverted when 400 nm light was used: Here, only 26 and 43% of C1V1-ET/ET animals responded, while 85 and 89% of ChR2-HR animals showed escape behavior. Thus, C1V1-ET/ET can robustly trigger neuronal activity, and does so more pronounced at green-shifted wavelengths, when compared to ChR2-HR.

### C1V1-ET/ET Activation in Muscles Counteracts ChR2-HR in GABAergic Motor Neurons

**Figure 3:**
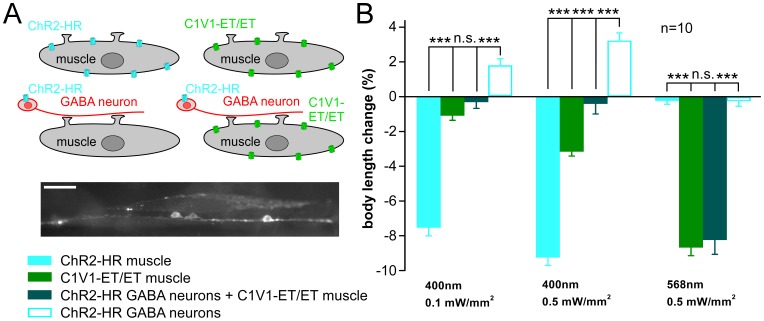
(Co-)expression of ChR2-HR and C1V1-ET/ET in different cell types of the same animal. **A)** ChR2-HR and C1V1-ET/ET were expressed in different cell types (GABAergic motor neurons or body wall muscle cells), either in different transgenic lines, or co-expressed in the same animal, as indicated in the pictograms. Fluorescence micrograph shows expression in the same animal of ChR2-HR::YFP in GABA neurons and C1V1-ET/ET::YFP in muscle cells. **B)** Body length changes (contraction or relaxation = elongation; given in relation to the initial body length of unstimulated animals) were measured in response to light of the indicated wavelengths and intensities. Residual contractions evoked by 400 nm light by C1V1-ET/ET in muscle could be counteracted, but not overcome, by ChR2-HR in GABA neurons. Displayed are mean body length changes and s.e.m. N = 10 animals of each strain were measured. Statistically significant differences were calculated by two-tailed Student’s t-test (n.s. = non significant; ***p<0.001).

GABAergic motor neurons innervate muscle cells (Fig. 3A) and evoke muscle hyperpolarization (and body relaxation of ∼4%), when photo-triggered [27]. Thus, ChR2-HR activation in these cells should counteract C1V1-ET/ET activation in muscle, which by itself evokes body contraction of ∼9% (Fig. 3B). When we compared nematodes expressing ChR2-HR::YFP in GABAergic neurons to animals expressing both C1V1-ET/ET::YFP in muscle cells and ChR2-HR::YFP in GABA neurons (Fig. 3A, B), we observed that at 400 nm, the effects of full GABA neuron activation and residual muscle activation (i.e. 400 nm still activating C1V1-ET/ET somewhat) antagonized each other, thus demonstrating that both rhodopsins could be specifically activated in different cell types of the same animal (Fig. 3B). Importantly, also in *C. elegans*, C1V1-ET/ET appears to have a higher light sensitivity when compared to ChR2-HR. Therefore, even at 400 nm, some C1V1-ET/ET is co-activated with ChR2-HR, thus preventing fully independent use of both tools. To overcome this limitation, we aimed at a ChR2 variant with high light sensitivity, to use lower light intensities for stimulation at 400 nm, which would not co-activate C1V1-ET/ET. Thus, we expressed the ChR2 mutant T159C (ChR2-TC) which was reported to be more efficient for stimulation with lower light intensities compared to ChR2-HR, without a spectral shift [19], in body wall muscle cells. Because from other experiments we had indications that the H134R mutation is beneficial for expression of ChR2 in the *C. elegans* background, we also expressed T159C as a double mutant together with H134R (ChR2-HR/TC) (Fig. 1B, C).

### ChR2-HR/TC Activation Evokes Contraction of *C. elegans* Muscle Cells with High Sensitivity in *vivo*

ChR2-TC::YFP as well as ChR2-HR/TC::YFP expressed well in body wall muscle cells, and largely localized to the plasma membrane, however, also forming some clusters in the cell interior (Fig. 1C). To better enable comparison of potential differences in effectiveness of the individual ChR2 variants, we roughly compared expression levels of the particular proteins based on YFP fluorescence of whole animals. Expression levels estimated this way depend on both the expression amount in single muscles, and, as we used non-integrated transgenes to compare these proteins, on the likelihood of achieving uniform expression in all muscles (i.e. low mosaicisms; this is generally the better, the more expression of a particular transgenic protein is tolerated). YFP fluorescence of ChR2-HR/TC was clearly higher than that of ChR2-HR, followed by ChR2-TC (Fig. S7). Compared to either of the other three proteins, C1V1-ET/ET::YFP expression levels were lowest.

In order to find a complementary blue-light dependent counterpart for C1V1-ET/ET activation, action spectra of the actuators were acquired, using 5 different wavelengths between 400 and 580 nm (Fig. 1B). Surprisingly, ChR2-TC animals could not be stimulated to maximal contractions (which lie at ca. 15%; [27]), at either wavelength. However, using ChR2-HR/TC, strong contractions of 15% could be evoked at 480 nm, compared to 12% *via* ChR2-HR. Peak activity of ChR2-TC compared to ChR2-HR was not altered, displaying a maximum between 440 and 480 nm, and no measurable activity at 580 nm under these conditions. In contrast, C1V1-ET/ET shows a red shifted activation spectrum, peaking at 520 nm within the applied wavelengths, yet with still some activity at 400 nm. (Fig. 1B). For better comparison, spectra were also normalized to the peak activity, showing statistically significant different contractions for C1V1-ET/ET and ChR2-HR/TC at 400 and 580 nm (Fig. S3). Importantly, when light intensities were fine-tuned to 0.01 mW/mm^2^ for 400 nm, and 0.2 mW/mm^2^ for 580 nm, excitation of both ChR2-HR/TC and C1V1-ET/ET expressed in body wall muscle cells in separate transgenic lines could be achieved with no cross activation of either protein using the respective off-peak light color (Fig. 1D).

### C1V1-ET/ET and ChR2-HR/TC Allow Independent Photoactivation of Two Classes of Neurons in the Same Animal

**Figure 4:**
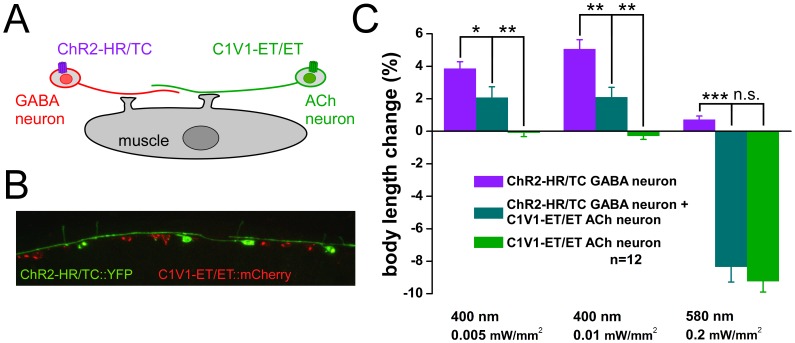
ChR2-HR/TC in GABA neurons and C1V1-ET/ET in ACh neurons enable independent bi-modal light control of either cell type. A) Innervation of muscle cells by GABAergic and cholinergic motor neurons, expressing ChR2-HR/TC and C1V1-ET/ET, respectively, in the same animal. **B)** Confocal fluorescence image of ChR2-HR/TC::YFP and C1V1-ET/ET::mCherry expressed in the ventral nerve cord motor neurons. **C)** Body length changes were measured in response to 400 or 580 nm light of the indicated intensities. Animals used expressed either ChR2-HR/TC in GABA neurons, or C1V1-ET/ET in cholinergic neurons, or both. When both proteins were expressed in the same animal, independent activation of both proteins and the respective neuron type can be achieved. Displayed are mean body length changes, relative to the initial length of the non-illuminated animals, and s.e.m. N = 12 animals of each strain were measured. Statistically significant differences were calculated by two-tailed Student’s t-test (n.s. = non significant; *p<0.05; **p<0.01; ***p<0.001).

The measured action spectra of the different channelrhodopsin variants tested here indicated that it should be possible to stimulate two different sets of neurons independently in the same animal, using different colors of light. To test this directly, we expressed ChR2-HR/TC in GABAergic motor neurons (from the p*unc-47* promoter), and C1V1-ET/ET in cholinergic motor neurons (from the p*unc-17* promoter), as independent activation of each neuron class should be obvious by their push-pull influence on body length (Fig. 4A). First, we tested each protein in the respective neurons independently, expressing only one or the other (Fig. 4C). In contraction assays, animals expressing ChR2-HR/TC in GABAergic motor neurons reacted with an elongation when stimulated with low intensities of 400 nm light (0.005 and 0.01 mW/mm^2^; Fig. 4C). In contrast, animals expressing C1V1-ET/ET in cholinergic motor neurons did not respond to such low intensity of violet light. However, upon stimulation with 580 nm light, C1V1-ET/ET animals responded with strong contractions of 9.2%, whereas ChR2-HR/TC evoked only little (0.7%) relaxation (Fig. 4C). Interestingly, in this setup, ChR2-HR/TC animals appeared to react more sensitive to yellow light than animals expressing ChR2-HR/TC directly in muscles. Finally, we assayed changes in body length evoked by photoactivation of ChR2-HR/TC and C1V1-ET/ET in animals co-expressing both, ChR2-HR/TC::YFP in GABAergic, and C1V1-ET/ET::mCherry in cholinergic motor neurons (Fig. 4A, B) under the different illumination conditions. Here, ChR2-HR/TC activation in GABAergic neurons with violet light caused moderate relaxation of 2%. In the same animals, C1V1-ET/ET in cholinergic neurons triggered strong contractions at 580 nm (Fig. 4C). The reduced effects of ChR2-HR/TC were due to the co-expression of C1V1-ET/ET in the same multicopy array, as co-injection of similar amounts of non-relevant DNA had similar reducing effects (Fig. S8); such negative effects may be prevented by generating separate arrays and crossing them. Overall, we hereby demonstrated that both channelrhodopsin variants, ChR2-HR/TC and C1V1-ET/ET, can be activated in parallel in the same animal, yet independently, using light of different wavelengths, to evoke independent signals to downstream cells.

## Discussion

In this work, we could establish two new channelrhodopsin variants, the green-light activated C1V1-ET/ET and the blue-light triggered ChR2-HR/TC, both with higher efficiency than ChR2-HR, for applications in *C. elegans*. These two ChRs can be combined to achieve independent excitation of two neuron types in the same animal. These tools will be very useful in multimodal analyses of neuronal circuits, in which two different nodes can be independently activated, while additional neurons may be inactivated using light driven ion pumps for hyperpolarization. See Table 1 for a summary of the relevant parameters determined for each of the proteins analyzed in this study.

**Table 1:** Properties of Channelrhodopsin variants tested in this study in *C. elegans* body wall muscle cells.

Protein	Expression in BWM	Light evoked contractions (%±sem)	Wavelength(nm)	Lines/tested/reacted	Rating
**ChR2-HR**	+++	−6.62±0.51 (Fig. 1A)−12.17±0.63 (Fig. 1B)	458480	>10/5/5	++
**ChR2-TC**	++	−6.17±0.49	480	>2/2/1	+
**ChR2-HR/TC**	++++	−15.08±0.56	480	>3/3/3	+++
**C1C2-5-2**	++	−5.14±0.70	480	>2/2/1	+
**vChR1 hum**	+	−0.85±0.26	540	>5/2/1	+/−
**vChR1 (trunc)**	+	−0.19±0.40	540	>2/2/0	–
**vChR1 short**	+	−0.49±0.42	540	>2/2/0	–
**vChR1 short SP**	++	−0.63±0.53	540	>3/2/0	–
**C1V1-2-5**	++	−3.78±0.92	540	>3/2/2	+
**C1V1-ET/ET**	++	−8.85±1.03	520	>4/2/2	++
**C1V1-ET/VI/GA**	++	−0.27±0.39	540	>4/3/0	–
**V1C2-2-2-3**	–	−1.37±1.56	540	>2/2/0	–

Summary of the results obtained with ChR::YFP fusion proteins expressed in body wall muscle (BWM) cells. Expression obtained from extrachromosomal transgenes were compared by eye, for expression level, localization to the plasma membrane and mosaic expression patterns, and classified from – (no expression) to +++ (robust, uniform and predominantly plasma membrane expression). Photo evoked contractions were deduced from work presented in the main and supplementary figures, given as relative body contraction in % of the initial length, measured at the indicated wavelength near the respective action spectrum maximum. The number of generated transgenic lines is given, as well as lines with appreciable expression, tested for light evoked reactions, and number of lines responding as by visual inspection. Each time, the best-responding line was further analyzed for quantitative data as presented throughout. ‘Rating’: A non-quantitative, arbitrary summary of the parameters tested for each protein, and from impressions obtained while experimenting with each; ranked from +++ (very well-working optogenetic tool, in *C. elegans*) to - (non-working optogenetic tool, in *C. elegans*).

C1V1-ET/ET expressed well in muscles and neurons of *C. elegans*, and enabled potent photoactivation of both cell types. C1V1-ET/ET could be fully stimulated with yellow light, where no activity of ChR2-HR was evoked. At 400 nm, it was only partially possible to activate ChR2-HR without some co-activation of C1V1-ET/ET. This is an intrinsic problem since all rhodopsins absorb some light at wavelengths shorter than the main absorption band (the ß-band). However, using a blue light absorbing ChR that expresses well and shows large currents, one can minimize these effects by adjusting the light intensity to moderate values. Combining the two well expressing ChR2 mutants T159C with H134R, we could achieve very good expression levels in muscles and neurons resulting in better reactivity with lower light intensities, well suited for our application in combination with C1V1-ET/ET. Fine-tuning the relative expression levels of the two double mutant proteins in motor neurons, we were able to further improve the differential excitation of two cell populations with 400 and 580 nm in the same animal.

Upon co-expression of ChR2-HR/TC in GABAergic and C1V1-ET/ET in cholinergic neurons, photo-triggered length changes were highly affected by the expression levels of the individual proteins. Thus, empirically varying the amount of injected DNA is required to achieve best results, as this will also depend on the respective promoter used for each protein and cell type. Maximal activity of ChR2-HR/TC expressed in GABAergic motor neurons was thus lowered due to the presence of other co-injected DNA in the array, lowering its effectiveness. In contrast, photoreactivity of animals expressing C1V1-ET/ET in cholinergic motor neurons was hardly influenced by accessory co-injected DNA. Such effects may also vary depending on the promoter.

Overall, C1V1-ET/ET and ChR2-HR/TC provide strong and valuable tools for independent dual color photostimulation of two cells or cell-types in *C. elegans*, which will allow to address different neurons or neuron classes functionally within the same circuit. Furthermore, using NpHR, Mac or Arch to mediate photoinhibition of even additional neurons, and combining these with a multicolor structured illumination and behavior tracking system that we recently developed [18], [33], as well as recombinase-based genetic methods for single-cell specific expression of optogenetic tools [36], will allow to analyze neuronal circuits in *C. elegans* with unprecedented accuracy and versatility. The development of red shifted optical excitation could also pave the way for utilization with simultaneous imaging using genetically encoded Ca^2+^ indicators (GECIs) based on GFP, CFP or YFP, particularly the ratiometric cameleons [34], [35]. This desired compatibility would allow for an all-optical approach to investigate direct downstream effects and connectivity. However, with the current residual activity of C1V1-ET/ET in the blue range of the spectrum, we could not yet achieve cameleon imaging without concomitant activation of C1V1-ET/ET, thus further red-shifting of the actuator, and/or blue-shifting of the sensor may be required. Combining ChR2 and the red-shifted, though non-ratiometric Ca^2+^ indicator RCaMP, however, allows such approaches already [37].

## Supporting Information

Figure S1 **Channelrhodopsin variants used or generated and tested in this study.** Color coding indicates the origin of the respective TM domains of the ChRs used, either from *Chlamydomonas* ChR1 or 2, or from *Volvox* VChR1. Point mutations are indicated by red ovals. For exact nature of the mutations, see Methods and main paper.
(PDF)

Figure S2 **VChR1 is not functionally expressed in** ***C. elegans*** **body wall muscle cells. A)** Several versions of VChR1::YFP (for description see Fig. S1 and Methods) were expressed in body wall muscle cells and fluorescent micrographs were obtained. **B)** Body contractions evoked by the indicated VChR1 variants at 540 nm light were compared to ChR2-HR evoked contractions at 458 nm light. Displayed are mean body length changes and s.e.m. N = number of animals of each strain tested is indicated. Statistically significant differences in mean animal length (in pixels) compared to the respective period before illumination, was determined by paired two-tailed Student’s t-test (***p<0.001; **p<0.01; n.s. = non significant).
(PDF)

Figure S3 **Comparison of action spectra of various ChRs tested in this work, related to** **Fig. 1A** **(left panel), and B (right panel).** Data as presented in Figure 1A and B were normalized to the peak contractions evoked for each protein tested, and statistically significant contractions for each protein and wavelength were compared to the respective contraction evoked by ChR2-HR as a reference, at the same wavelength, by two-tailed Student’s t-test (***p<0.001; **p<0.01; *p<0.05, n.s. = non significant).
(PDF)

Figure S4 **Action Spectra of the tested proteins in HEK 293T cells or** ***C. elegans*** **muscle cells.** **A)** Action spectra of tested channelrhodopsin variants recorded in whole-cell patch clamp measurements on HEK 293T cells. Spectra were linearly normalized on the light intensity at each wavelength. Table summarizes the fitted peaks of the action spectra; sometimes, two peaks were observed. **B)** Action spectra measurements based on evoked body contractions in *C. elegans* muscle cells. Body length changes were measured in videos of animals that were filmed for 3 sec before a light stimulus of the indicated wavelength was presented for 2 sec, and then the relaxation was recorded for another 3 sec. The mean body length changes were averaged for the indicated periods before and during the light stimulus, and these values were used to construct the action spectra. Light intensities were adjusted for each wavelength.
(PDF)

Figure S5 **Expression and functionality of C1V1, C1V1-ET/VI/GA and V1C2-2-2-3 in** ***C. elegans*** **muscle cells. A)** Fluorescent micrographs of the indicated proteins expressed in muscle. **B)** Body length changes evoked by 540 nm light to the indicated number of animals expressing each of the three proteins. Significance compared to mean pixel values before illumination after paired two-tailed Student’s t-test (***p<0.001).
(PDF)

Figure S6 **Escape responses in mechanosensory neurons, evoked by C1V1 or ChR2-HR.** Upper panel: Touch receptor neurons of *C. elegans.* Lower panel: Escape responses of N = 20 animals were compared for the two proteins at the indicated wavelengths, as described in the main paper, Fig. 2. Significance calculated after two-tailed Student’s t-test (***<0.001).
(PDF)

Figure S7 **Visual Comparison of expression levels of Channelrhodopsin variants in** ***C. elegans*** **muscle.** Fluorescent micrographs of animals expressing the indicated protein::YFP fusions in body muscle cells.
(PDF)

Figure S8 **Effects of co-injection of DNA on transgene levels and evoked behaviors.** The animals analyzed in Fig. 4 of the main paper were generated by injecting different amounts of the respective DNA for ChR2-HR/TC and C1V1-ET/ET. To better estimate the effects of one DNA being present in the same extrachromosomal array on the abundance of the other DNA (and thus the evoked effects by the respecticve protein), control strains were generated in which the same amount of DNA of non-relevant sequence (DNA ladder, see Methods) was co-injected. The presence of non-relevant DNA in the transgene reduces the expression and effects evoked by the respective ChR. Significance calculated after two-tailed Student’s t-test (***p<0.001).
(PDF)
