# Supplementary figures and images for: Bimodal Activation of Different Neuron Classes with the Spectrally Red-Shifted Channelrhodopsin Chimera C1V1 in Caenorhabditis elegans
Source: PLoS One. 2012 Oct 3;7(10):e46827. doi: 10.1371/journal.pone.0046827 (PMC3463556; doi:10.1371/journal.pone.0046827)

Figure S1

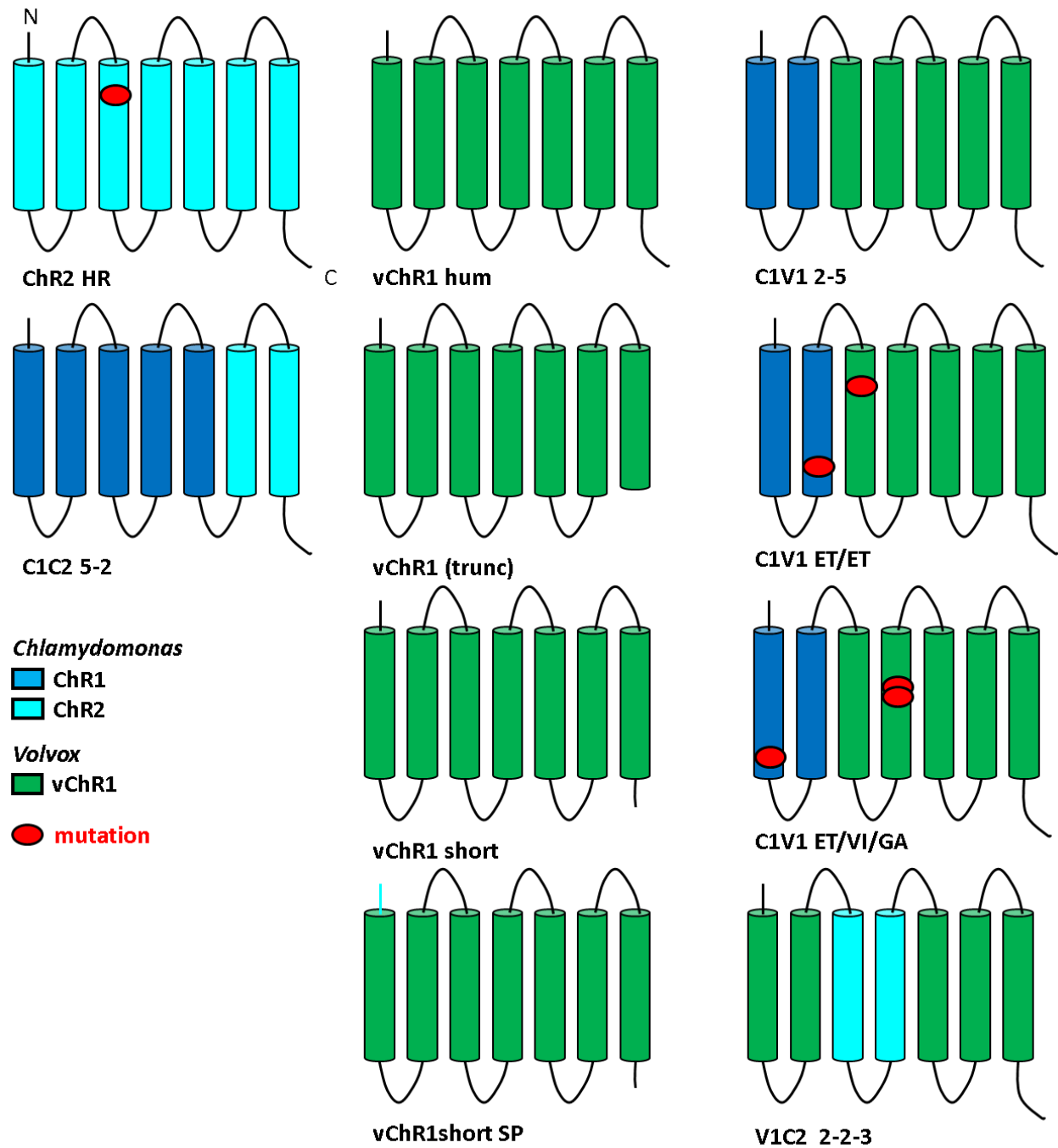

Supplement: Figure S1 — Channelrhodopsin variants used or generated and tested in this study. Color coding indicates the origin of the respective TM domains of the ChRs used, either from Chlamydomonas ChR1 or 2, or from Volvox VChR1. Point mutations are indicated by red ovals. For exact nature of the mutations, see Methods and main paper. (PDF) [file pone.0046827.s001.pdf]

Figure S2

A

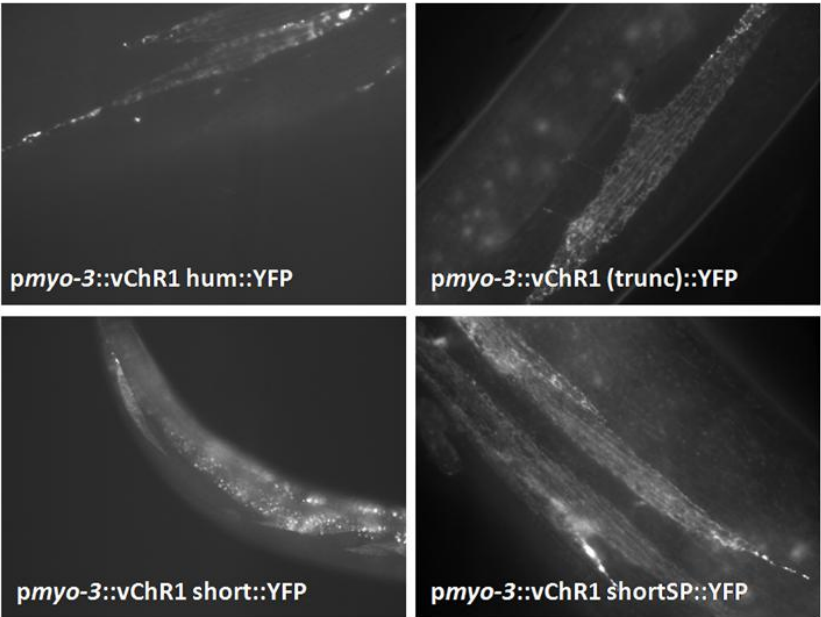

B

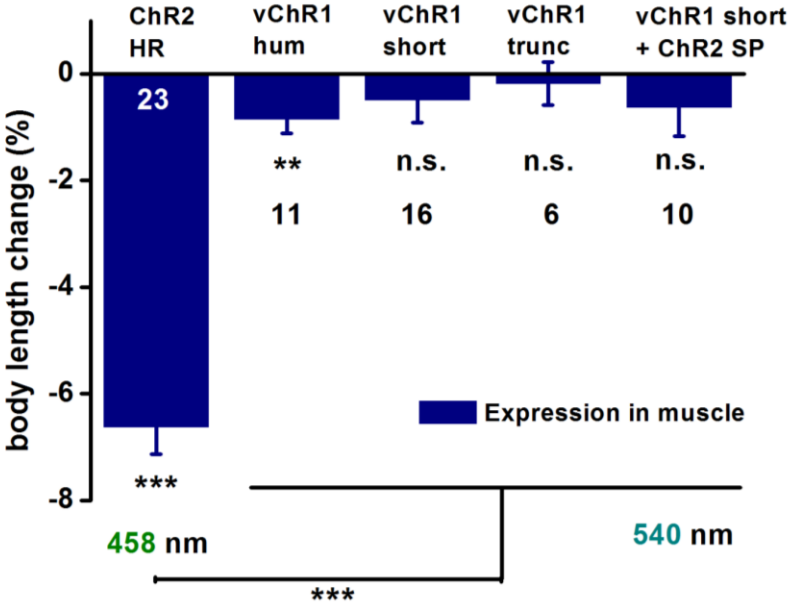

Supplement: Figure S2 — VChR1 is not functionally expressed in C. elegans body wall muscle cells. A) Several versions of VChR1::YFP (for description see Fig. S1 and Methods) were expressed in body wall muscle cells and fluorescent micrographs were obtained. B) Body contractions evoked by the indicated VChR1 variants at 540 nm light were compared to ChR2-HR evoked contractions at 458 nm light. Displayed are mean body length changes and s.e.m. N = number of animals of each strain tested is indicated. Statistically significant differences in mean animal length (in pixels) compared to the respective period before illumination, was determined by paired two-tailed Student’s t-test (***p<0.001; **p<0.01; n.s. = non significant). (PDF) [file pone.0046827.s002.pdf]

Figure S3

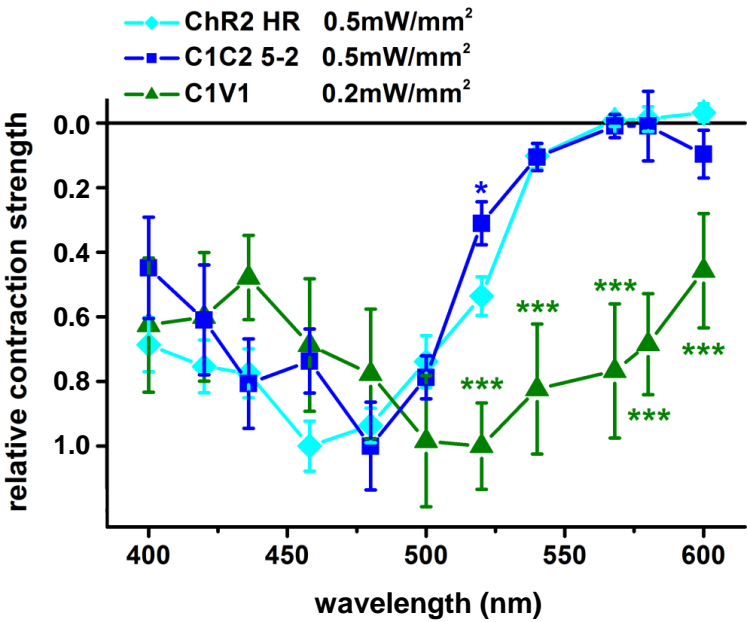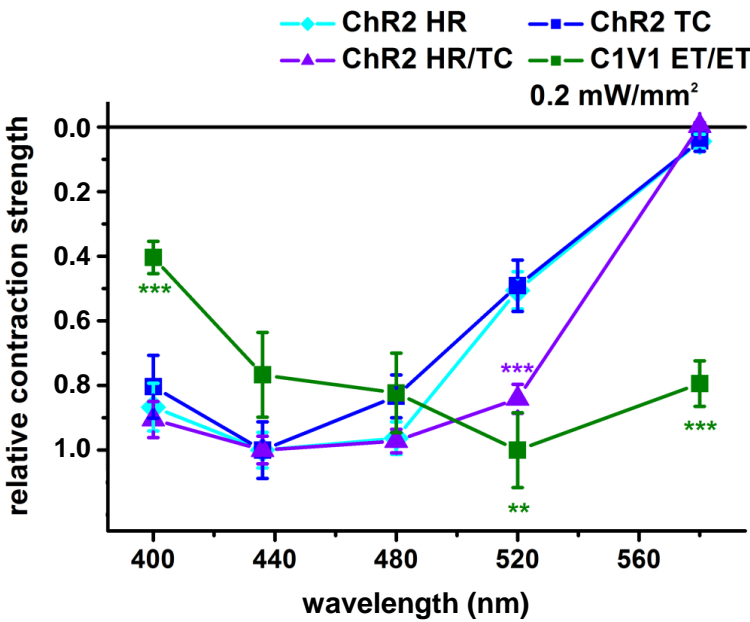

Supplement: Figure S3 — Comparison of action spectra of various ChRs tested in this work, related to Fig. 1A (left panel), and B (right panel). Data as presented in Figure 1A and B were normalized to the peak contractions evoked for each protein tested, and statistically significant contractions for each protein and wavelength were compared to the respective contraction evoked by ChR2-HR as a reference, at the same wavelength, by two-tailed Student’s t-test (***p<0.001; **p<0.01; *p<0.05, n.s. = non significant). (PDF) [file pone.0046827.s003.pdf]

Figure S4

A

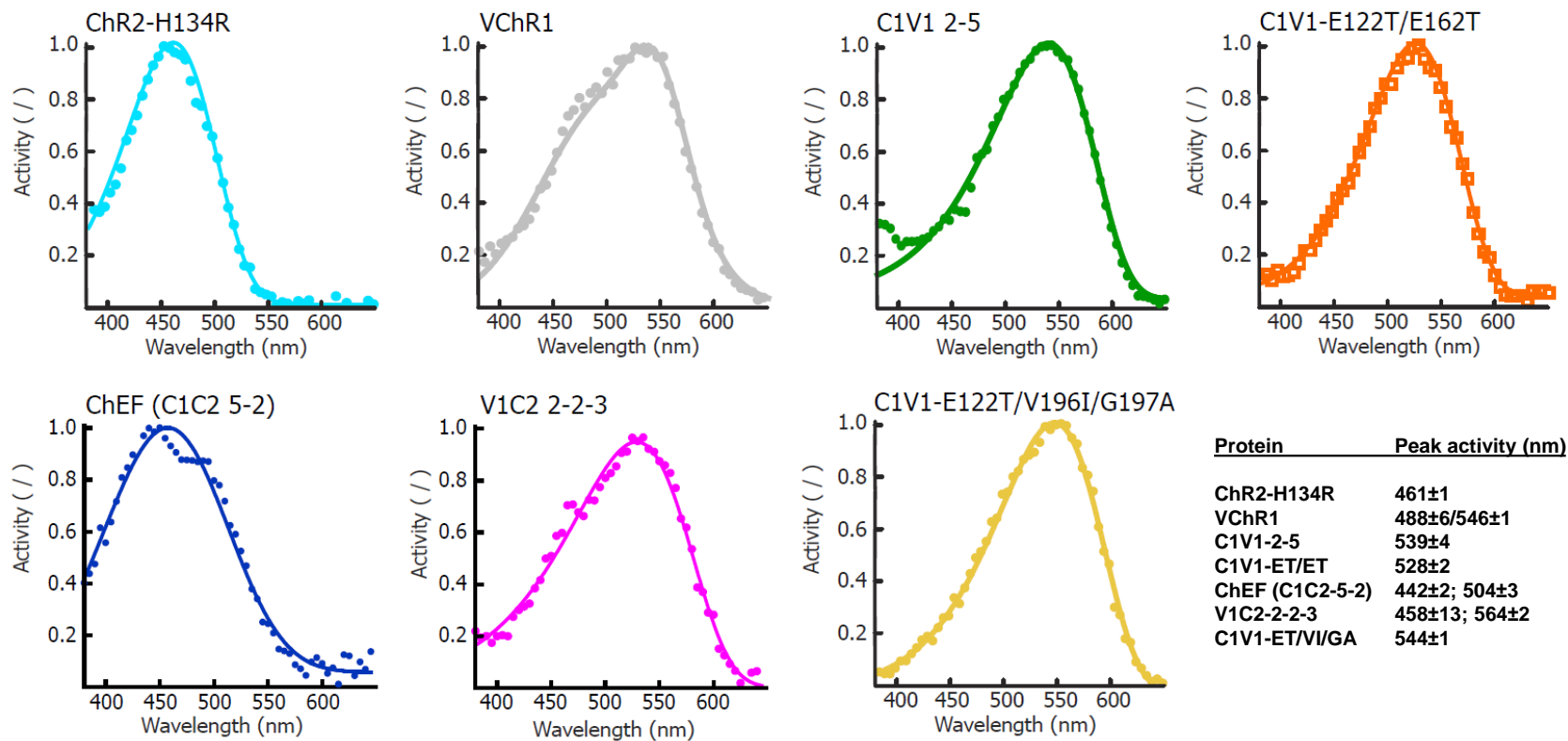

B

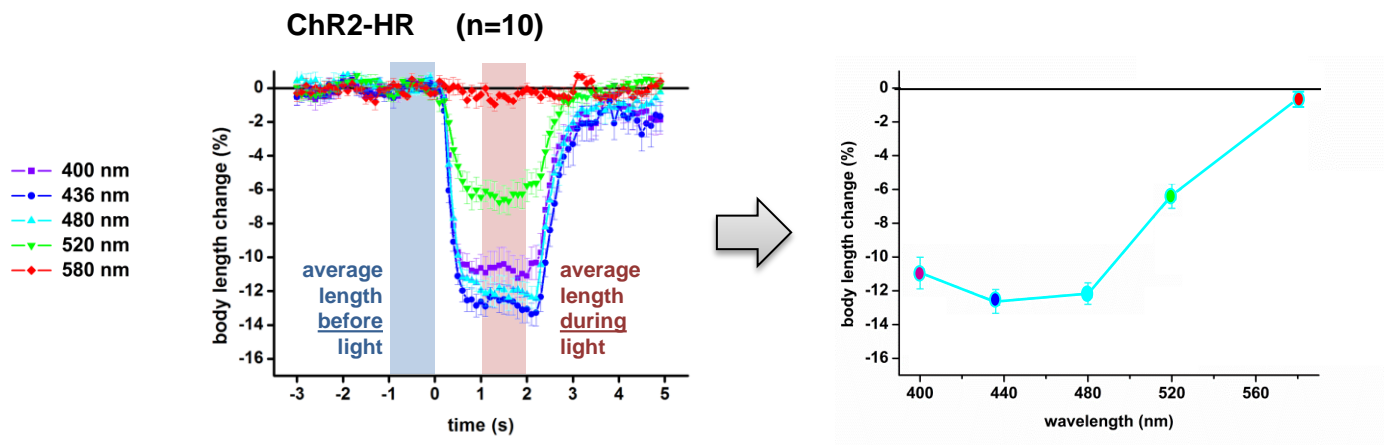

Supplement: Figure S4 — Action Spectra of the tested proteins in HEK 293T cells or C. elegans muscle cells. A) Action spectra of tested channelrhodopsin variants recorded in whole-cell patch clamp measurements on HEK 293T cells. Spectra were linearly normalized on the light intensity at each wavelength. Table summarizes the fitted peaks of the action spectra; sometimes, two peaks were observed. B) Action spectra measurements based on evoked body contractions in C. elegans muscle cells. Body length changes were measured in videos of animals that were filmed for 3 sec before a light stimulus of the indicated wavelength was presented for 2 sec, and then the relaxation was recorded for another 3 sec. The mean body length changes were averaged for the indicated periods before and during the light stimulus, and these values were used to construct the action spectra. Light intensities were adjusted for each wavelength. (PDF) [file pone.0046827.s004.pdf]

Figure S5

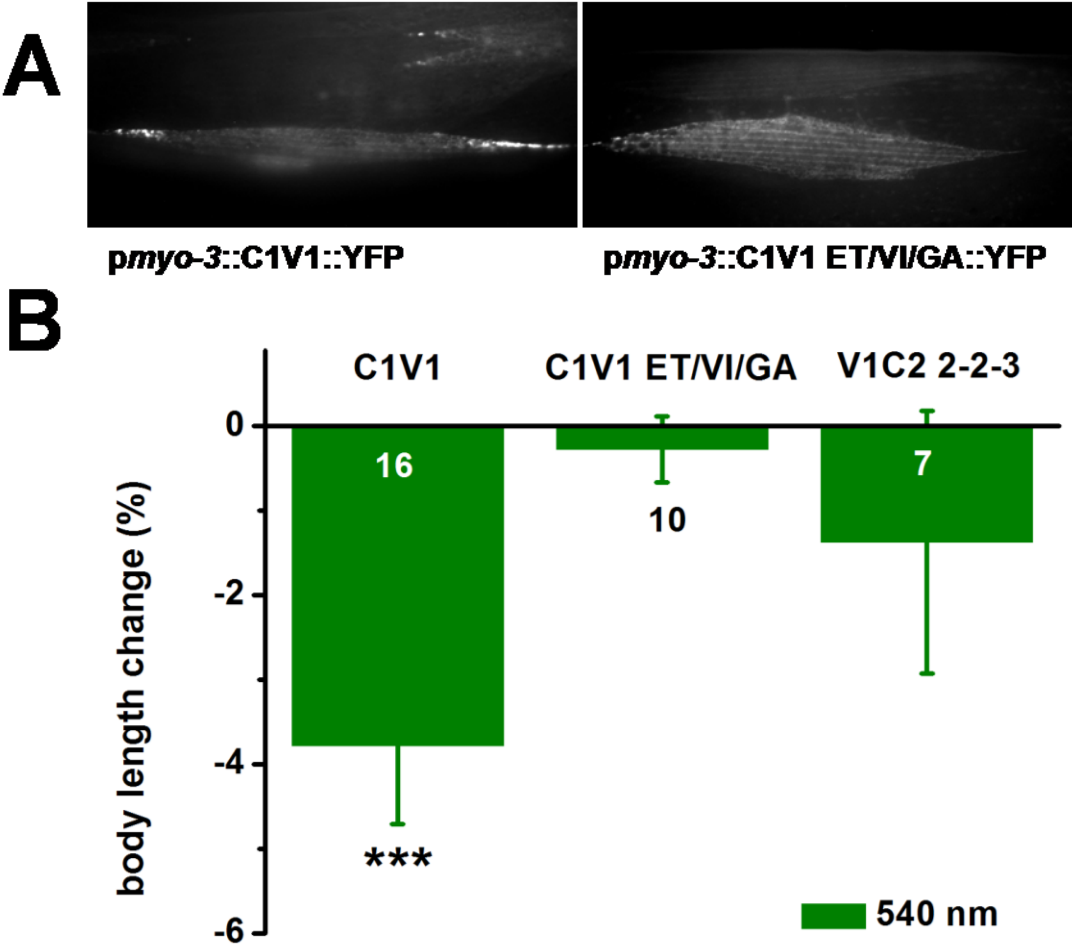

Supplement: Figure S5 — Expression and functionality of C1V1, C1V1-ET/VI/GA and V1C2-2-2-3 in C. elegans muscle cells. A) Fluorescent micrographs of the indicated proteins expressed in muscle. B) Body length changes evoked by 540 nm light to the indicated number of animals expressing each of the three proteins. Significance compared to mean pixel values before illumination after paired two-tailed Student’s t-test (***p<0.001). (PDF) [file pone.0046827.s005.pdf]

Figure S6

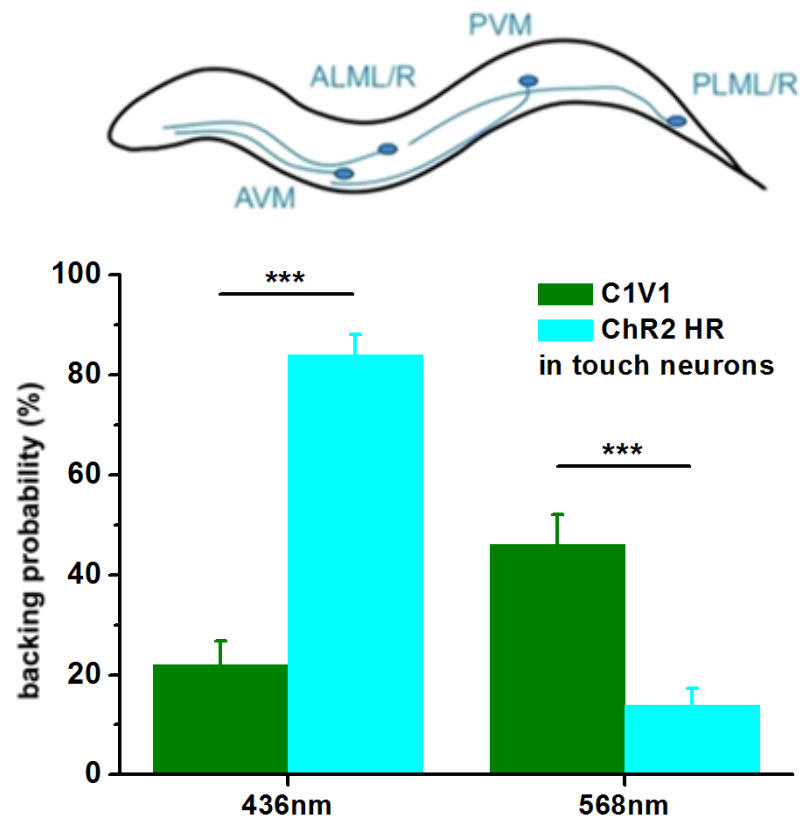

Supplement: Figure S6 — Escape responses in mechanosensory neurons, evoked by C1V1 or ChR2-HR. Upper panel: Touch receptor neurons of C. elegans. Lower panel: Escape responses of N = 20 animals were compared for the two proteins at the indicated wavelengths, as described in the main paper, Fig. 2. Significance calculated after two-tailed Student’s t-test (***<0.001). (PDF) [file pone.0046827.s006.pdf]

Figure S7

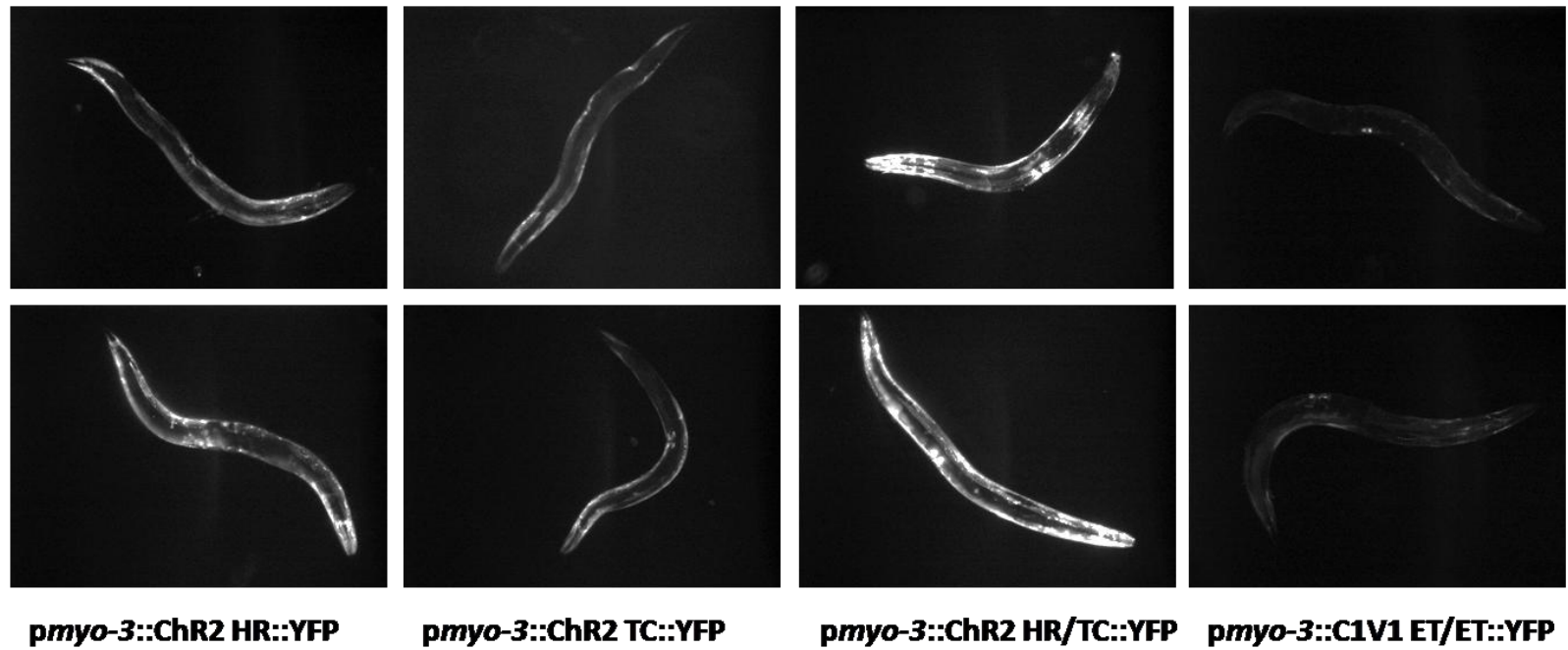

Supplement: Figure S7 — Visual Comparison of expression levels of Channelrhodopsin variants in C. elegans muscle. Fluorescent micrographs of animals expressing the indicated protein::YFP fusions in body muscle cells. (PDF) [file pone.0046827.s007.pdf]

Figure S8

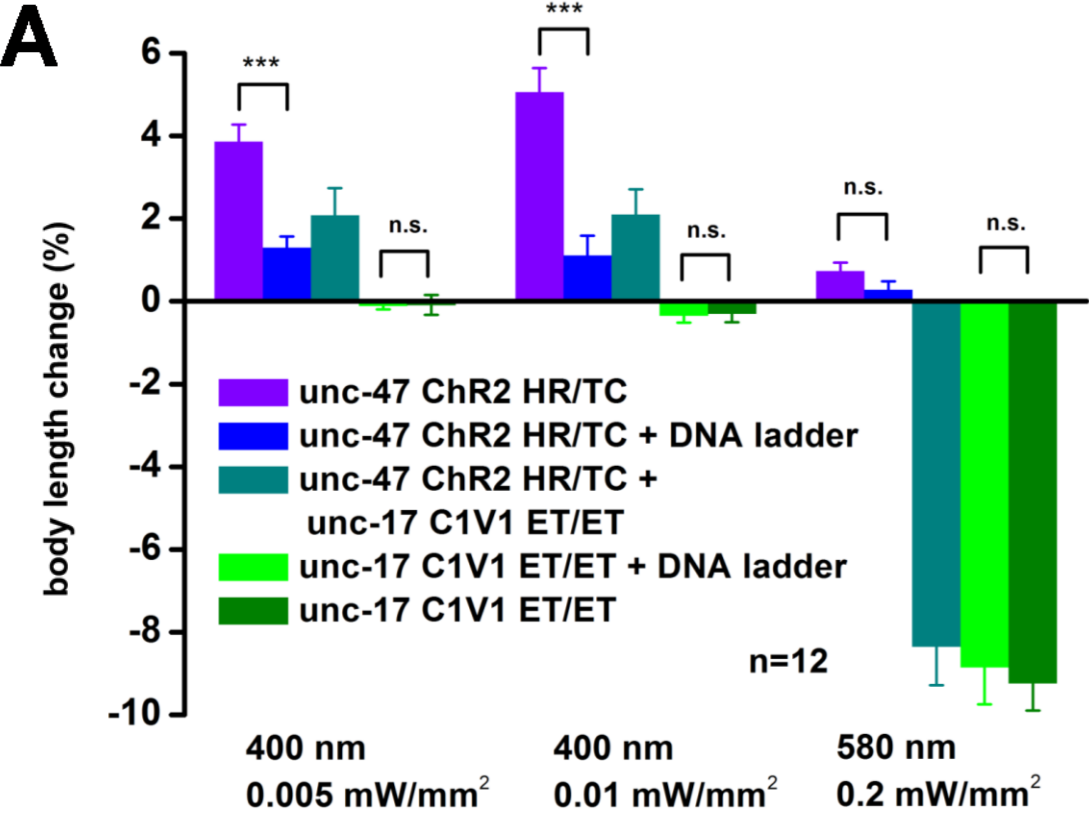

Supplement: Figure S8 — Effects of co-injection of DNA on transgene levels and evoked behaviors. The animals analyzed in Fig. 4 of the main paper were generated by injecting different amounts of the respective DNA for ChR2-HR/TC and C1V1-ET/ET. To better estimate the effects of one DNA being present in the same extrachromosomal array on the abundance of the other DNA (and thus the evoked effects by the respecticve protein), control strains were generated in which the same amount of DNA of non-relevant sequence (DNA ladder, see Methods) was co-injected. The presence of non-relevant DNA in the transgene reduces the expression and effects evoked by the respective ChR. Significance calculated after two-tailed Student’s t-test (***p<0.001). (PDF) [file pone.0046827.s008.pdf]
